# Identifying Distinct Latent Profiles of Executive Functioning Among Adolescents and Adults With Anorexia Nervosa and Adolescent Healthy Controls

**DOI:** 10.1002/erv.70043

**Published:** 2025-10-23

**Authors:** Jiana Schnabel, Marita Cooper, Kristin Stedal, Mark Rose, Betteke Maria van Noort, Deborah A. G. Drabick, Lauren B. Alloy, C. Alix Timko

**Affiliations:** ^1^ Department of Psychology and Neuroscience Temple University Philadelphia Pennsylvania USA; ^2^ Department of Child and Adolescent Psychiatry and Behavioral Sciences Children's Hospital of Philadelphia Philadelphia Pennsylvania USA; ^3^ Regional Department of Eating Disorders Division of Mental Health and Addiction Oslo University Hospital Ullevål HF Oslo Norway; ^4^ Dorset HealthCare University NHS Foundation Trust Dorset UK; ^5^ Department of Psychology MSB Medical School Berlin Berlin Germany; ^6^ Department of Psychiatry Perelman School of Medicine University of Pennsylvania Philadelphia Pennsylvania USA

**Keywords:** adolescents, anorexia nervosa, development, executive functioning, latent profile analysis

## Abstract

**Objective:**

Research suggests executive functioning (EF) inefficiencies contribute to anorexia nervosa (AN) onset and maintenance. Identifying EF subtypes in adolescents and adults with AN compared to healthy controls (HC) may provide insights into differences in illness severity, risk for prolonged illness, and highlight who could respond best to different treatments.

**Method:**

We conducted secondary analysis of 751 participants: adolescents (*n* = 559) and adults with AN (*n* = 74), and adolescent HC (*n* = 118). Latent profiles of six Delis Kaplan Executive Function System scores measuring EF constructs implicated in AN were derived. Differences across profiles on demographics, eating disorder cognitions (EDE/EDE‐Q score), BMI/BMI *z*‐score, length of illness, weight suppression, and full‐scale IQ were examined.

**Results:**

A three‐profile solution best fit the data: Profile 1 (*n* = 324)—‘high verbal’, Profile 2 (*n* = 349)—‘average’, and Profile 3 (*n* = 78)—‘low flexibility and inhibition’. The low flexibility and inhibition profile comprised 10.6% of adolescents with AN, 30.0% of adults with AN, and 1.7% of adolescent HCs. Compared to other profiles, this profile was older, had a longer illness duration, higher EDE global scores, lower BMI *z*‐scores, and lower full‐scale IQ scores. No profile differences emerged in BMI or EDE‐Q global scores.

**Discussion:**

Only a small subset of our sample showed marked difficulties in flexibility and inhibition, challenging the notion that EF difficulties are a core feature of AN. Adolescents with AN in this profile showed greater illness severity, suggesting vulnerability to a more prolonged course of illness. These findings are a first step towards developing tailored treatment strategies based on EF profiles.

## Highlights

- We identified three distinct executive functioning profiles among adolescents and adults with anorexia nervosa (AN) and adolescent healthy controls: Profile 1 (‘high verbal’), Profile 2 (‘average’), and Profile 3 (‘low flexibility and inhibition’).
- The low flexibility and inhibition profile, comprising only 12% of the AN sample and containing the highest proportion of adults across profiles, was associated with greater illness severity and duration, challenging the idea that executive functioning inefficiencies are a universal endophenotype of the disorder.
- These results represent a first step towards developing tailored treatment strategies based on executive functioning profiles.

## 1 Introduction

Difficulties in certain components of executive functioning (EF) are implicated in the aetiology and maintenance of anorexia nervosa (AN; Bartholdy et al. 2016; Brockmeyer et al. 2022; Kucharska et al. 2019). Data from adult samples overarchingly suggest weaknesses in cognitive flexibility and central coherence play a role in acute and remitted AN (Miles et al. 2020; Stedal et al. 2021), leading to the hypothesis that inefficiencies in these components of EF represent an endophenotype of the disorder (Pappaianni et al. 2023; Tenconi et al. 2010). By contrast, studies in youth show a modest difference in EF, at most, between those with AN and healthy controls (HC; Lang et al. 2014; Stedal et al. 2022; Westwood et al. 2016). Several hypotheses may explain these divergent findings: EF inefficiencies in adults could be a consequence of malnutrition resulting from the illness (i.e., a scar), could indicate risk for a persistent course or symptom maintenance (Rodgers et al. 2023; Smith et al. 2018), AN onset might disrupt EF development during adolescence, or methodological differences in assessing EF across studies may contribute to inconsistent results (Miles et al. 2022). Given the considerable heterogeneity in EF findings, particularly regarding which domains are affected and in whom, there is a need to investigate potential EF^1^ subtypes within AN populations using standardised, person‐centred approaches across the lifespan.

EF reaches full maturity between adolescence and early adulthood (Boelema et al. 2014; Huizinga et al. 2006; Korzeniowski et al. 2021; Lee et al. 2013). This sensitive period of EF development is particularly relevant in AN, where the mean age at treatment presentation is 15 years (Timko et al. in preparation). Although most available evidence suggests adolescents with AN exhibit only minor weaknesses in EF, neurocognitive inefficiencies are more pronounced in older adolescent patients, possibly due to a longer duration of illness (Stedal et al. 2022). Greater severity of eating pathology and lower BMI also are associated with difficulties in cognitive flexibility and central coherence in adolescents with AN (Lozano‐Serra et al. 2014), which may reflect the role of EF as a maintenance factor or even a complication related to more severe malnutrition. Indeed, data from low‐ and middle‐income countries has shown impairments in EF development following prolonged malnutrition (Kirolos et al. 2022). To better understand the role of EF in the aetiology of AN, research focussing on differences between adolescents and adults with AN may contribute to our understanding of how malnutrition impacts EF development.

Although studies often examine individual components of EF, such as flexibility or central coherence, in isolation, more recent conceptualizations indicate the need to understand EF as both unique and integrated processes (Karr et al. 2022). Rose et al. (2016) attempted to study EF holistically via hierarchical cluster analysis to identify discrete neuropsychological profiles in adolescents with AN and HC. Their results revealed three clusters in adolescents with AN: a neuropsychologically low to average cluster, a verbal and visuospatial discrepancy cluster, and a high‐performance verbal and neuropsychologically average cluster. These results contrasted the clusters found in HC, where one HC cluster demonstrated poor visuospatial memory but high verbal fluency, and a second cluster exhibited average performance on all neuropsychological tasks. This study provides initial evidence of the neuropsychological heterogeneity underlying AN.

Although cluster analysis is a practical exploratory step, the method has significant limitations. Cluster analysis (e.g., k‐means clustering), a non‐model‐based approach, relies on optimisation algorithms to minimise within‐cluster and/or maximise between‐cluster variation, using distance or dissimilarity metrics that are sensitive to variable scaling and multicollinearity (Ketchen and Shook 1996). These methods lack model fit statistics and probabilistic estimates of class membership, making model selection more arbitrary, less formally guided, and more reliant on researchers' subjective decisions (Magidson and Vermunt 2002; Xu et al. 2013). Latent profile analysis (LPA) offers several advantages over cluster analytic techniques, all of which aim to decrease heterogeneity by identifying meaningful subgroups within a sample. As a model‐based approach, LPA hypothesises a mixture of probability distributions, uses maximum likelihood estimation, and includes a variety of statistical tests to identify the best fitting model (Magidson and Vermunt 2002; Xu et al. 2013), thereby reducing researcher bias and increasing the accuracy of the analysis (Berlin et al. 2014). As a person‐centred approach, LPA categorises individuals into relatively uniform groups, with members more similar to each other than to those in other groups. This allows researchers to detect distinct patterns of characteristics within a population, offering a more robust and objective approach to subgroup identification than cluster analysis. LPA recently has been used in AN research to identify personality‐based profiles (Di Lodovico et al. 2024), assess weight gain trajectory during treatment, and examine clinical symptoms and treatment outcomes across profiles (Wade et al. 2020).

Despite the utility of LPA in identifying subgroups in individuals with AN, only one study has used LPA to examine EF profiles in adolescents with AN and HC (Drake 2019). They identified five profiles in adolescents with AN, which were characterised by global difficulties, memory weakness, average performance, visual‐verbal discrepancy, and high average with strong verbal abilities. These profiles varied by age and BMI, with younger individuals with higher BMIs clustering in the high‐average with strong verbal profile and older participants with lower BMIs in the global difficulties profile. These findings provide tentative support for the clinical utility of neuropsychologically derived subgroups of AN. For HC, four profiles emerged: memory weakness, above‐average, discrepant performance, and strong verbal, indicating variability in cognitive strengths and weaknesses among healthy individuals. However, one drawback of Drake's study was that AN profiles only were compared within the AN group, whereas HC profiles only were compared within the HC group, preventing direct comparisons between AN and HC profiles.

This study builds on the work of Drake (2019) by applying LPA to selected scores from the Delis‐Kaplan Executive Function System (D‐KEFS) in a combined sample of adolescents and adults with AN and adolescent HC. The aim is to identify distinct EF profiles and determine whether these profiles differ in demographic and clinical characteristics. Including all groups in a single LPA allows for direct comparison of shared and unique cognitive profiles across different stages of the disorder. This approach potentially highlights differences between more recent‐onset cases (adolescents) and those with a persistent course (adults) while also providing insights into the impact of illness duration and malnutrition on EF profiles. Additionally, it helps evaluate whether EF inefficiencies are an endophenotype of AN and clarifies which EF difficulties are specific to the disorder. Based on the results from previous studies assessing neuropsychological profiles in individuals with AN and HC (Rose et al. 2016; Schnabel, Stedal, et al. 2025), we hypothesised the presence of at least two profiles: one that reflects more difficulty inhibiting cognitive interference and cognitive flexibility, and another reflecting moderate to high performance on all included measures of EF. We anticipated most adults with AN would fall into the lower performance profile. In contrast, we hypothesised that most adolescent HC would fall into the moderate to high‐performance profile, while adolescents with AN would be split between the profiles. We also predicted the profiles would be clinically distinct, such that profiles characterised by poorer EF performance would be associated with greater illness severity, reflected in higher EDE or EDE‐Q scores, lower BMI or BMI *z*‐scores, higher weight suppression, and longer length of illness, compared to higher‐performing profiles.

## 2 Methods

### 2.1 Participants

The sample comprised 751 participants (559 adolescents with AN, 74 adults with AN, and 118 adolescent HC) from two separate cohorts (see Table 1). We categorised participants by age based on the normative ranges specified in the D‐KEFS technical manual: adolescents (8–19 years) and adults (20–49 years and 50–89 years) (Delis et al. 2001). Cohort A consisted of all adults with AN, the adolescent HC, and 312 adolescents with AN, drawn from a dataset of 606 individuals participating in an international multi‐site study investigating neuropsychological functioning using the Ravello Profile (Stedal, Frampton, et al. 2012). The Ravello Profile is a neuropsychological test battery (administered in a standardised order) specifically developed to assess areas of functioning that previous research has indicated may be atypical in patients with AN (Rose et al. 2011). A subset of these participants was included in a prior study that examined neuropsychological clusters in adolescents with AN and HC (Rose et al. 2016). Cohort B included 247 adolescents with AN, recruited from three clinical trials and a longitudinal observational study conducted at the Children's Hospital of Philadelphia's eating disorders programme across inpatient and outpatient settings. All participants met the inclusion criteria for an AN diagnosis, including subclinical and atypical AN presentations. Subclinical AN was defined as meeting all diagnostic criteria for AN except for the degree of weight loss (i.e., not losing > 20% of body weight), whereas atypical AN was defined as having a BMI that was not objectively low for sex and age (i.e., BMI percentile > 90% of median body weight). Participants in Cohort A were diagnosed according to DSM‐IV criteria if aged 17 or older, or via the Great Ormond Street (GOS) Diagnostic Checklist if aged 16 or younger, given its superior reliability in younger populations (Nicholls et al. 2000). Diagnoses were retrospectively updated to meet DSM‐5 criteria. Participants in Cohort B were diagnosed according to DSM‐5 criteria. Qualified clinicians at the respective hospital or clinic site made all diagnoses. Additionally, all participants were required to have two or more relevant D‐KEFS achievement scores and to be assessed upon treatment engagement, to ensure that only baseline assessments, and not repeated assessments from the same individuals, were included. As a result, 102 of the 606 data points from the Ravello dataset were excluded from the analyses. Ethics approvals were obtained for all original studies. The current study, which used anonymised data, was deemed exempt from human subject review.

**TABLE 1:** Cohort characteristics.

	Cohort A	Cohort B
Study sites	Ravello profile	Children's Hospital of Philadelphia
Sample size (*n*)	504	247
Countries	Scotland	United States of America
	Norway	
	Germany	
	England	
Diagnostic age groups	Adolescents with AN: 312	Adolescents with AN: 247
	Adults with AN: 74	
	Adolescent healthy controls: 118	
Age range	Adolescents with AN: 9–19	Adolescents with AN: 10–19
	Adults with AN: 20–52	
	Adolescent healthy controls: 9–19	
Diagnosis	DSM IV criteria	DSM 5 criteria
	AN‐R: 334	AN‐R: 205
	AN‐BP: 27	AN‐BP: 20
	Subclinical AN: 25	Subclinical AN: 3
	AAN‐R: 0	AAN‐R: 16
	AAN‐BP: 0	AAN‐BP: 3
	HC: 118	HC: 0
Demographic data	Age	Age
	Sex	Sex
	Race/ethnicity	Race/ethnicity
D‐KEFS subtests	Trail making test: Number–letter switching	Same as Cohort A
	Trail making test: Motor speed	
	Verbal fluency: Category switching	
	Verbal fluency: Category switching accuracy	
	Colour–word interference: Inhibition	
	Colour–word interference: Inhibition/switching	
Clinical variables	BMI	BMI *z*‐score
	BMI *z*‐score	Full scale IQ
	Full scale IQ	EDE‐Q global score
	EDE global score	Length of illness
		Developmental weight suppression

Abbreviations: AAN, atypical anorexia nervosa; AN, anorexia nervosa; AN‐BP, anorexia nervosa binge‐purge subtype; AN‐R, anorexia nervosa restricting subtype; D‐KEFS, Delis‐Kaplan Executive Function System; EDE, Eating Disorder Examination interview; EDE‐Q, Eating Disorder Examination Questionnaire; HC, healthy control.

### 2.2 Measures

#### 2.2.1 Demographic and Eating Disorder‐Related Variables

Demographic information, including age, biological sex, race, ethnicity, diagnosis, and weight status (BMI/BMI *z*‐score), was available for all participants. BMI *z*‐scores, which are validated for individuals aged 2–19 years, were calculated for adolescent participants in our sample. For Cohort B, illness severity measures, including weight suppression and length of illness, were collected.

#### 2.2.2 Delis Kaplan Executive Function System (D‐KEFS; Delis et al. 2001)

The D‐KEFS is a comprehensive, standardised battery of nine assessments of EF that can be used with individuals ages 8–89. It has been validated for use with individuals with AN (Fitzpatrick et al. 2012). The subtests in the Ravello Profile include the Trail Making Test, Verbal Fluency Test, Colour‐Word Interference Test (Condition 3: Inhibition; Condition 4: Inhibition/Switching), and the Tower Test. Cohort B was not assessed using the full Ravello profile, but completed the same measures from the Trail Making Test, Verbal Fluency Test, and Colour‐Word Interference Test. These adolescents also were administered the Sorting Test in place of the Tower Test.

The present study included scaled scores from the Trail Making Test Condition 4 (Number Letter Switching), which measures cognitive flexibility by requiring participants to connect alternating numbers and letters in sequential order (e.g., 1–A–2–B–3–C) under timed conditions, and Condition 5 (Motor speed), which measures graphomotor processing speed by having participants connect numbers in sequence as quickly as possible. From the Verbal Fluency Test, we included Condition 3 (Category Switching: Total Correct), which assesses the ability to shift between semantic categories (e.g., naming fruits and then animals), and Condition 4 (Category Switching: Total Switching Accuracy), which assesses accuracy during these shifts. From the Colour‐Word Interference Test, we used Condition 3 (Inhibition), which requires participants to name the ink colour of a word while ignoring the printed word itself (e.g., the word ‘red’ printed in blue ink), and Condition 4 (Inhibition/Switching), which alternates between naming ink colours and reading words based on contextual cues. Together, these subtests assess cognitive flexibility, verbal fluency, inhibition, and motor processing speed, aspects of EF with established empirical support for being inefficient or enhanced in AN (Kjaersdam Telléus et al. 2015; Stedal, Frampton, et al. 2012). Our assessment battery did not include a dedicated measure of working memory, a component of EF (Miyake et al. 2000). However, to maintain consistency with prior work, we selected many of the same subtests used in previous studies examining neuropsychological performance in AN (Drake 2019; Rose et al. 2016).

#### 2.2.3 Intelligence Assessment

Intelligence was measured using the Wechsler scales in Switzerland, Norway, and the UK, and the Culture Fair Intelligence Test–20–Revision (CFIT‐20; Weiß 2006) in Germany. IQ scores were estimated using the Vocabulary and Matrix Reasoning subtests from either the Wechsler Abbreviated Scale of Intelligence (WASI; Wechsler 1999) or the Wechsler Adult Intelligence Scale–Third Edition (WAIS‐III; Wechsler 1997). For Cohort B, all participants completed the two‐subtest version of the Wechsler Abbreviated Scale of Intelligence–Second Edition (WASI‐II; Wechsler 2011). This study used IQ scores from the two subtest versions of the WASI and WASI‐II to calculate a Full‐Scale Intelligence Quotient (FSIQ‐2).

#### 2.2.4 Eating Disorder Examination and Eating Disorder Examination Questionnaire (EDE or EDE‐Q; Fairburn and Cooper 1993; Fairburn and Beglin 1994)

To assess eating disorder cognitions, participants in Cohort A completed the EDE, and Cohort B completed the EDE‐Q. Both have four subscales (restraint, eating concern, shape concern, and weight concern) and an overall global score, with higher scores indicating greater symptom severity. The internal consistency of the EDE Global score (Cronbach's *α* = 0.88) and EDE‐Q Global score (Cronbach's *α* = 0.93) in the present sample was good.

### 2.3 Statistical Analyses

Estimating power for LPA is inherently complex, as it requires knowledge of population parameters such as profile proportions, means, variances, and covariances, values that often rely on prior research or theoretical assumptions, which may not always be available (Spurk et al. 2020). Consequently, we did not conduct a formal power analysis. However, simulation studies by Nylund et al. (2007) and Spurk et al. (2020) suggest that a minimum sample size of approximately 500 is generally sufficient for accurately identifying latent profiles. The optimal sample size depends on model complexity, with more indicators typically requiring larger samples for reliable results. In our study, we included six indicators. Prior research has demonstrated that three profile solutions can be detected with six indicators in samples of 431 (Kapantzoglou et al. 2015) and 520 (Orri et al. 2017), while four profile solutions have been identified with six indicators in samples of 764 (Chen et al. 2017) and 656 (Ren et al. 2019). These findings suggest that our sample size of 751 was appropriate for the current study.

We combined Cohorts A and B for analysis. All analyses were conducted in R Studio (v4.3.2; R Core Team 2023). Of 4506 observations, 374 data points were missing at random (MAR; data collection from Cohort B was impacted by the COVID‐19 pandemic related shutdowns). Given the low levels of missing data, richness of the dataset in terms of constructs measured using multiple levels of analysis, and ability to estimate scores based on observed data (MAR assumption), we used single imputation under a Gaussian finite mixture model, applying an expectation–maximisation algorithm to estimate missing values based on observed data to predict missing values based on the available data. We performed latent profile analysis to identify the optimal number of latent profiles (Rosenberg et al. 2018). There is no single ‘gold standard’ for determining the best model fit (Drabick et al. 2021). LPA modelling begins by specifying a single profile and incrementally adding profiles until there is no meaningful improvement in model fit (Nylund et al. 2007). We evaluated one‐ to four‐profile solutions using criteria described by Bauer (2022): (1) Bayesian information criterion (BIC) and Akaike's information criterion (AIC); lower scores on these indices indicate a better model fit; (2) Entropy, which indexes how well profiles are separated from each other (≥ 0.80 is preferred); (3) Bootstrap likelihood ratio test (BLRT), which evaluates the fit of a model with *k* classes to the model with *k*‐1 classes; (4) Profile size; profiles containing < 5%–10% of the sample suggest overfitting of the model to the data, which could limit generalisability (Ferguson et al. 2020); and (5) Parsimony and group meaningfulness.

Following identifying the optimal number of profiles, we conducted Chi‐Square (*χ* ^2^) tests to compare profiles in terms of the number of adolescents with AN, adults with AN, and adolescent HC, as well as sex differences. We also evaluated profile differences in demographic characteristics and measures of illness severity (age, EDE/EDE‐Q global score, BMI/BMI *z*‐score, length of illness, weight suppression, and FSIQ‐2) via Analysis of Variance (ANOVA), or Kruskal‐Wallis tests where parametric assumptions were not met. Normality was assessed using visual inspection and the Kolmogorov–Smirnov test, whereas homogeneity of variance was evaluated using Levene's test. We applied multiple comparison corrections to the main effect results using a Bonferroni‐Holm *p* value adjustment at an alpha level of 0.05 (Holm 1979). After Bonferroni‐Holm *p*‐value corrections, significant main effects were explored further using the Tukey‐Kramer method for ANOVA or pairwise Mann‐Whitney *U* comparisons for Kruskal‐Wallis Tests. Effect sizes were calculated using Cramer's V or eta squared (*η* ^2^) for categorical and continuous data. Effect sizes for *η* ^2^ were classified as small 0.01, medium 0.06, and large 0.14, whereas the strength of effect size for Cramer's V is small 0.05, medium 0.15, and large 0.22 (Cohen 1988).

## 3 Results

Demographic characteristics of the sample are presented in Table 2.

**TABLE 2:** Demographic characteristics of sample.

	Adolescents with AN	Adults with AN	Adolescent HC
Sample size	559	74	118
Age (years)	15.39 (SD = 1.91)	26.32 (SD = 5.95)	15.09 (SD = 2.14)
Sex	515 (92.1%) females	73 (98.6%) females	116 (98.3%) females
Race		74 (100%)	118 (100%)
White	529 (86.1%)		
Black	8 (1.4%)		
Native American	1 (0.2%)		
Asian	10 (1.8%)		
Other race	7 (1.3%)		
Unreported	4 (0.7%)		
Ethnicity	15 (2.7%) Hispanic	0% Hispanic	0% Hispanic
Diagnosis			
AN‐R	481 (86.1%)	58 (78.4%)	
AN‐BP	32 (5.7%)	15 (20.3%)	
Subclinical AN	27 (4.8%)	1 (1.4%)	
AAN R	16 (2.9%)		
AAN BP	3 (0.5%)		
Control			118 (100%)
BMI *z*‐score	−1.56 (SD = 1.50)		0.27 (SD = 0.83)
BMI		15.26 (SD = 2.14)	
Profile membership			
Profile 1—High verbal	243 (43.5%)	28 (37.8%)	53 (44.9%)
Profile 2—Average	257 (45.9%)	29 (39.2%)	63 (53.4%)
Profile 3—Low flexibility and inhibition	59 (10.6%)	17 (30.0%)	2 (1.7%)

Abbreviations: AAN‐BP, atypical anorexia binge purge subtype; AAN‐R, atypical anorexia restricting subtype; AN‐BP, anorexia nervosa binge purge subtype; AN‐R, anorexia nervosa restricting subtype; HC, healthy controls.

### 3.1 Latent Profile Analysis

**FIGURE 1:**
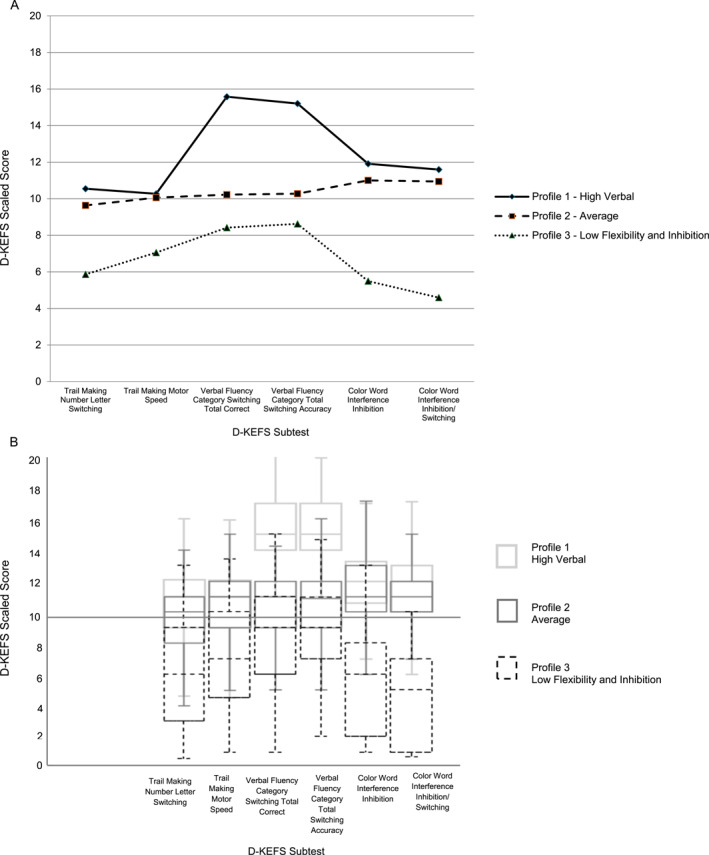
(A) D‐KEFS scaled scores by profile (line graph). (B) D‐KEFS scaled scores by profile (boxplots).

Fit indices for latent profile models with one‐ to four‐profile solutions are presented in Table 3. The four‐profile solution had slightly lower BIC and AIC values than the three‐profile solution. However, the four‐profile model included one profile with a small proportion of participants (5.7%), and profiles containing less than 10% of the sample may suggest overfitting of the data and can limit generalisability of the findings (Ferguson et al. 2020). Additionally, in the four‐profile model, the largest profile was split into two profiles with similar means and patterns, making the three‐profile model the more parsimonious choice (Figure 1).

**TABLE 3:** Fit statistics for latent profile analyses.

Profiles	LL	BIC	AIC	Entropy	Smallest class %	BLRT *p*‐value
1	−11669.98	23419.41	23363.96	1.0	100%	N/A
2	−11279.87	22685.54	22597.73	0.74	43.68%	0.01
**3**	**−10981.29**	**22134.73**	**22014.57**	**0.82**	**10.39%**	**0.01**
4	−10885.15	21988.81	21836.31	0.84	5.72%	0.01

*Note:* Bold font represents the selected model.

Abbreviations: AIC, Akaike Information Criteria; BIC, Bayesian Information Criteria; BLRT, Bootstrap Likelihood Ratio Test; LL, log likelihood.

Analyses supported a three‐profile solution as achieving optimal fit and parsimony. Profile 1 (43.1%)—‘high verbal’ was characterised by the highest scores in verbal fluency and average scores on the Trail Making Test (Conditions 4 and 5) and Colour Word Interference Test (Conditions 3 and 4). Profile 2 (46.5%)—‘average’ represented the largest proportion of the sample and was characterised by average performance according to D‐KEFS standardised norms across all six subtests. Profile 3 (17.3%)‐ ‘low flexibility and inhibition’ was characterised by low average scores in verbal fluency and motor speed, and significantly lower scores on the Trail Making Test (Condition 4) and Colour Word Interference Test (Conditions 3 and 4). The means and standard deviations of each D‐KEFS subtest by profile are shown in Table 4.

**TABLE 4:** Means and standard deviations of D‐KEFS subtests scaled scores by profile.

	Profile 1 high verbal		Profile 2 average		Profile 3 low flexibility and inhibition	
	*M*	SD	*M*	SD	*M*	SD
Trail making test number letter switching	10.55	2.71	9.64	2.49	5.86	3.57
Trail making test motor speed	10.27	3.14	10.06	2.69	7.05	3.60
Verbal fluency category switching total correct	15.58	2.05	10.23	2.07	8.42	3.25
Verbal fluency category switching total switching accuracy	15.21	2.01	10.28	2.12	8.63	2.90
Colour word interference inhibition	11.91	2.14	11.00	2.13	5.50	3.44
Colour word interference inhibition/Switching	11.60	2.17	10.94	2.11	4.59	3.11

### 3.2 Comparison of Profiles on Demographic and Eating Disorder Characteristics

Characteristics of profiles and test statistics are in Table 5. Analyses revealed significant differences in the number of adolescents with AN, adults with AN, and adolescent HC across profiles (*p* < 0.001). Adults with AN were overrepresented in the low flexibility and inhibition profile compared to the high verbal and average profiles; adolescent HC were underrepresented in the low flexibility and inhibition profile, in comparison to the high verbal and average profiles. The distribution of males and females also significantly differed across profiles (*p* = 0.002), with the low flexibility and inhibition profile including a relatively higher proportion of males than the other profiles. However, given the very low base rate of males in the sample, this finding should not be overinterpreted. Group differences across markers of clinical severity were mostly significant but small. We found profiles differed significantly in terms of age, with older participants in the low flexibility and inhibition profile compared to the high verbal profile (*p* < 0.001) and average profile (*p* = 0.001). There were also differences in EDE global scores across profiles. Individuals in the low flexibility and inhibition profile had significantly higher EDE Global scores than the high verbal (*p* = 0.008) and average (*p* < 0.001) profiles. Differences in BMI *z*‐score were found across profiles, with the low flexibility and inhibition profile having significantly lower BMI *z*‐scores than the average profile (*p* = 0.015). Profiles differed on length of illness, with the high verbal profile having a significantly shorter duration of illness compared to the average (*p* = 0.021) and the low flexibility and inhibition (*p* = 0.048) profiles. There were significant differences between profiles on FSIQ‐2 score. FSIQ‐2 scores were higher in the high verbal profile than in the average (*p* < 0.001) and low flexibility and inhibition (*p* < 0.001) profiles. FSIQ‐2 scores in the average profile also were higher than in the low flexibility and inhibition profile (*p* < 0.001). However, we found no significant differences in BMI, EDE‐Q global scores, or weight suppression across profiles.

**TABLE 5:** Eating disorder characteristics of profiles.

	Profile 1 high verbal	Profile 2 average	Profile 3 low flexibility and inhibition	Test statistic	*p*	Effect size
				Chi‐square (*χ* ^2^)		*V*
Diagnostic age group						
Adults AN	28 (8.6%)	29 (8.3%)	17 (21.8%)	*χ* ^2^ (4) 22.56	0.000	0.123
Adolescents AN	243 (75.0%)	257 (73.6%)	59 (75.6%)			
Adolescent HC	53 (16.4%)	63 (18.1%)	2 (2.5%)			
Sex	10 (3.1%) males	27 (7.7%) males	10 (12.8%) males	*χ* ^2^ (2) 12.58	0.002^a^ ^,^ ^b^	0.092

				Kruskal Wallis		*p*	*η* ^2^
				*H*	*df*		
Age	16.11 (SD = 3.82)	16.27 (SD = 3.81)	18.40 (SD = 6.34)	15.32	2	0.000^b^ ^,^ ^c^	0.020
BMI *z*‐score	−1.26 (SD = 1.44)	−1.13 (SD = 1.66)	−1.61 (SD = 1.63)	7.56	2	0.022^c^	0.010
noEDE global	2.83 (SD = 1.80)	2.35 (SD = 1.73)	3.55 (SD = 1.67)	20.66	2	0.000^b^ ^,^ ^c^	0.042
EDE‐Q global	2.93 (SD = 1.80)	2.94 (SD = 1.73)	2.68 (SD = 1.98)	0.34	2	0.843	0.001
Length of illness (months)	10.55 (SD = 14.10)	13.20 (SD = 15.71)	15.40 (SD = 17.70)	6.78	2	0.033^a^ ^,^ ^b^	0.024
Developmental weight suppression	1.35 (SD = 0.74)	1.46 (SD = 0.99)	1.26 (SD = 0.82)	1.12	2	0.570	0.004

				ANOVA		*p*	*η* ^2^
				*F*	*df*		
BMI	15.91 (SD = 1.79)	15.81 (SD = 2.13)	15.84 (SD = 1.99)	0.10	2	0.890	0.001
WASI FSIQ‐2	111.77 (SD = 12.49)	105.44 (SD = 13.76)	95.07 (SD = 15.00)	46.77	2	0.000^a^ ^,^ ^b^ ^,^ ^c^	0.130

^a^ Significant post‐hoc differences between profile 1 and 2.

^b^ Significant post‐hoc differences between profile 1 and 3.

^c^ Significant post‐hoc differences between profile 2 and 3.

## 4 Discussion

The first aim of this study was to identify latent profiles of EF in a large sample (*N* = 751) of adolescents and adults with AN, as well as adolescent HC. Profile 1 (*n* = 324) exhibited very high verbal fluency scores and average cognitive flexibility, psychomotor speed, and inhibition scores. Profile 2 (*n* = 349) displayed average scores on all subtests, whereas Profile 3 (*n* = 78) displayed low average scores on the verbal measures and motor speed, and markedly lower scores in flexibility and inhibition. Notably, only a small subset of our sample, approximately 12% of individuals with AN, fell into the low cognitive flexibility and inhibition profile. This finding directly challenges the prevailing notion that EF inefficiencies are a core, universal endophenotype of AN, and instead suggests considerable heterogeneity in neurocognitive functioning among individuals with the disorder.

Identifying a three‐profile solution is consistent with findings from Renwick et al. (2015) and Rose et al. (2016), who also found three distinct neurocognitive clusters among individuals with AN. Importantly, we effectively replicated Rose and colleagues' findings using a larger sample that included a subset of their participants, which explains the similarity in results. Cluster 3 in Rose et al.’s study and Profile 1 (high verbal) are characterised by strong verbal performance alongside average to high average performance on other neuropsychological tasks, suggesting a consistent pattern of enhanced verbal fluency among a subset of individuals with AN. One possible explanation is that enhanced verbal fluency results from IQ strengths and especially strong verbal skills in a subgroup of patients with AN (Stedal, Rose, et al. 2012). Cluster 1 in Rose and colleagues' study exhibits low scores on the Trail Making Test and Colour Word Interference Conditions 3 and 4, which mirrors Profile 3 (low flexibility and inhibition).

Regarding inhibition, most studies show that performance on inhibition tasks continues developing until 15 years of age (Huizinga et al. 2006; Lee et al. 2013), except Colour Word Interference/Stroop tasks in which functional gains in efficiency continue to refine into early adulthood, up to age 21 (Leon‐Carrion et al. 2004). Participants in the low flexibility and inhibition profile have significantly lower scores on both Condition 3 and 4 of this subtest. Condition 4 of the Colour Word Interference Test is more demanding than Condition 3, as it requires both inhibition and cognitive switching, which may reveal a more subtle cognitive weakness not detectable by Condition 3 alone. Therefore, malnutrition during early adolescence, when AN typically onsets, may impact the development of inhibitory control in adults with AN such that it may not have matured to full capacity. Given the older age and higher percentage of adults in the low flexibility and inhibition profile, poorer performance on Colour Word Interference Conditions 3 and 4 could highlight the potential long‐term consequences of malnourishment on cognitive functioning in AN.

The second aim of the study was to explore differences in demographic and clinical variables across profiles. The significant sex differences observed across profiles suggest that males with AN may exhibit distinct patterns in cognitive functioning. However, given the small number of male participants, these findings should be interpreted with caution. Future research should replicate these findings with larger, more balanced samples to better understand potential sex‐specific patterns. Although all profiles include a mix of adolescents and adults with AN and adolescent HC, the low flexibility and inhibition profile had a distinctive composition, including only two HC (2.5%), suggesting that lower EF performance, specifically in terms of cognitive flexibility and inhibition, may be specific to individuals with AN. Considering that the low flexibility and inhibition profile also comprised the highest percentage of adults with AN (21.8%), membership in this profile may suggest EF inefficiencies are associated with risk for a more persistent course or a complication of prolonged length of illness. These results complement previous research suggesting that adults with AN are more frequently found to have abnormally low scores on EF measures compared to adolescents with AN or HC (Schnabel, Stedal, et al. 2025). Perhaps unsurprisingly, we also saw significantly older age and duration of illness in the low flexibility and inhibition profile, compared with the other profiles, given that age is highly correlated with and may be a proxy for length of illness (Grau et al. 2019) in adult samples, particularly when length of illness data are lacking. Our findings partially corroborate the results of the Stedal et al. meta‐analyses (2021, 2022), which found that EF difficulties were more pronounced in adults than adolescents, and that poorer EF performance was negatively associated with age. Similarly, we found that adolescents with a shorter duration of illness performed better on EF measures, highlighting the importance of early intervention to help prevent or minimise the effects of AN on EF.

In line with our predictions, we also found significant differences in clinical data such as EDE global score, BMI *z*‐score, and Full‐Scale IQ score across profiles. Findings regarding BMI‐z score and EDE global score are consistent with findings from Lozano‐Serra et al. (2014). Although IQ often is considered a stable trait, previous research shows that a drastic loss in body weight is associated with a decrease in performance on IQ measures in patients with AN (Ogata et al. 2021), and that there are significant improvements in performance on IQ measures after weight restoration (Schnabel, Jang, et al. 2025). In our sample, individuals in the low flexibility and inhibition profile also had the lowest BMI *z* scores, highest EDE global scores, and the longest illness duration, suggesting that IQ performance may represent a proxy for illness severity rather than premorbid ability. Therefore, we cannot determine participants' premorbid IQ and do not view IQ as a primary explanatory factor for profile membership.

Although there were differences across most demographic and clinical variables, no differences in BMI, weight suppression, or EDE‐Q global score were observed between profiles. BMI is a measure of weight status for individuals over 20; there were no significant differences in weight status in adults across the three profiles. However as noted above, we did find a difference in BMI *z*‐score across adolescents in each profile. Due to ongoing neurodevelopment, this may suggest that adolescents are more susceptible to cognitive difficulties caused by malnutrition from AN compared to adults. Interestingly, there were no differences in developmental weight suppression (DWS; the difference between an individual's highest premorbid BMI *z*‐score and current BMI *z*‐score), which is more sensitive to developmental factors such as age, height, and sex (Singh et al. 2021). Some research suggests that weight suppression may be linked to eating disorder symptoms and treatment outcomes (Sing et al. 2021); the current results imply that it may not be related to EF in AN. Nonetheless, adolescence is a period of rapid growth and brain development in which variations in weight status and body composition may have a more pronounced impact on cognitive functioning than in adulthood. In the context of AN, malnutrition may limit the brain's ability to capitalise on this neuroplasticity (Galler et al. 2017; Pizzol et al. 2021), reinforcing the need to continue examining how malnutrition impacts the development and differentiation of EFs.

Some assessment procedures differed by cohort, with Cohort A completing the EDE while Cohort B completed the EDE‐Q. Literature examining the relationship between the EDE and the EDE‐Q suggests that although both instruments measure similar constructs related to eating disorder cognitions, they have distinct characteristics and generate different levels of symptom severity. As a result, researchers and clinicians generally advise against using the two instruments interchangeably (Berg et al. 2011; Reas et al. 2011); thus, our samples were smaller when examining differences in eating disorder symptomology across profiles. The significant differences in EDE scores but the non‐significant EDE‐Q scores across profiles could be explained by variation in how well each measure captures eating disorder pathology. These assessments have been found to miss symptomatology in up to a third of individuals hospitalised with AN (Vanzhula et al. 2024). Future research should supplement the EDE or EDE‐Q with other assessments of AN symptoms [e.g., Eating Pathology Symptoms Inventory (Forbush et al. 2013)].

Our findings contradict suggestions of EF inefficiencies as a universal endophenotype for AN. The association between illness severity and EF performance, alongside a greater representation of adults in the low flexibility and inhibition profile, raises two possibilities: (1) EF inefficiencies represent a risk factor for a more persistent course of illness and/or (2) EF inefficiencies may be a consequence/scar of malnourishment from AN developing during adolescence. Because this study is cross‐sectional, it is not possible to determine whether EF inefficiencies are predictors or consequences of illness severity and long illness duration. Future longitudinal research throughout different stages of illness and recovery are essential to parse these links and examine the stability of these adolescent EF profiles over time. Characterising the progression of AN in the context of developing EF is essential for improving models of AN and our understanding of which adolescents will experience a persistent course of illness. However, there remains a lack of consensus regarding thresholds for impairment on the D‐KEFS and other cognitive tasks commonly used in clinical research. Establishing such thresholds (Schnabel, Stedal, et al. 2025) is necessary to recognise subgroups of patients who could benefit from more targeted or adjunctive treatments. For example, individuals in the low flexibility and inhibition profile who exhibit inefficiencies in these domains may benefit from adjunctive treatments such as Cognitive Remediation Therapy (Timko et al. 2024).

Our study has several strengths. To our knowledge, this is the first study to examine latent profiles across adolescents with AN, adults with AN, and adolescent HC. Including adolescents and adults with AN allowed us to use cross‐sectional data to explore how EF profiles may differ across developmental groups and in those with varying lengths of illness. Our sample size exceeds 500 strengthening the validity of our findings. The multi‐site international collaboration to contribute data allows for more generalisability of our results. Furthermore, we accounted for illness severity measures, such as length of illness and weight suppression, which are essential factors in understanding EF in AN but are not always included in studies of EF.

Our findings should be considered within the context of study limitations. First, in accordance with open science principles, we note that this study was not pre‐registered. Although pre‐registration is increasingly recognised as a best practice for transparency and rigour, it was not part of our standard procedures at the time of study initiation. Next, despite our large overall sample, only 10% were adults with AN, and 16% were HC, potentially limiting the generalisability of our comparison between groups. Similarly, most of our sample identified as cisgender female, White, and non‐Hispanic, with a diagnosis of AN‐R. Although preliminary data have not suggested differences in EF across restricting and binge‐purge subtypes, future studies should endeavour to replicate findings in a more diverse and representative sample. Additionally, the original studies did not include adult HC participants. Future research should conduct latent profiles of EF and include adolescent and adult participants, both with and without AN, to better distinguish illness effects from developmental factors. Another limitation is the absence of data on autistic traits, which are increasingly recognised as over‐represented in AN (Inal‐Kaleli et al. 2025) and may contribute to overlapping performance on cognitive tasks (e.g., Westwood et al. 2016). Including validated measures of autistic traits in future studies will help clarify their role in neurocognitive heterogeneity within AN. We also did not assess whether participants had previously completed the D‐KEFS or similar EF assessments, which are subject to practice effects; however, prior exposure is unlikely given their typical use in clinical evaluations for conditions that were largely exclusionary (e.g., developmental disability). Finally, although our study is a promising initial step in clarifying the role of cognitive flexibility and inhibition in AN, these EF domains cannot be considered in isolation. Our assessment battery did not include a dedicated measure of working memory, a component of EF that has shown significant differences between individuals with AN and HC in prior work (Stedal, Rose, et al. 2012). Therefore, future research should examine additional facets of EF (e.g., working memory) and other related factors of AN (e.g., personality, social/emotional factors, reward and punishment sensitivity) in combination with cognitive flexibility and inhibition inefficiencies to more holistically understand the aetiology of AN.

The primary focus of this study was to identify distinct neuropsychological profiles that exist in adolescents and adults with AN compared to adolescent healthy controls and examine differences among profiles. We found three distinct EF profiles that differ in demographic and illness severity measures, raising the question of whether malnutrition resulting from AN, particularly during adolescence, may have long‐term implications for EF. Our results challenge the notion that inefficiencies in EF are a universal endophenotype, and we propose that adolescents in Profile 3, the low flexibility and inhibition profile, are at higher risk for having a longer course of illness into adulthood or are already exhibiting the impact of malnourishment on EF development. This cautious proposition implies that the poorer performance in EF observed in this subgroup could contribute to the persistence or exacerbation of AN symptoms over time. Elucidating distinct profiles of EF was a positive first step in identifying those at risk for a prolonged illness course and beginning to tailor interventions accordingly. By acknowledging the strengths and weaknesses in different groups of individuals' profiles, early identification of adolescents exhibiting lower EF performance may facilitate targeted interventions that address cognitive inefficiencies and mitigate the risk of a protracted illness trajectory.

## Author Contributions

**Jiana Schnabel:** conceptualization, writing – original draft, writing – review and editing, data curation, formal analysis. **Marita Cooper:** conceptualization, writing – original draft, writing – review and editing, formal analysis. **Kristin Stedal:** investigation, writing – review and editing. **Mark Rose:** investigation, writing – review and editing. **Betteke Maria van Noort:** investigation, writing – review and editing. **Deborah A. G. Drabick:** writing – review and editing. **Lauren B. Alloy:** writing – review and editing. **C. Alix Timko:** conceptualization, funding acquisition, investigation, writing – original draft, writing – review and editing.

## Conflicts of Interest

The authors declare no conflicts of interest.

## Supporting information


**Table S1**: Means and Standard Deviations of D‐KEFS Subtests Scaled Scores by Diagnostic Age Group.

## Data Availability

The datasets generated during and/or analysed during the current study are available from the corresponding author upon reasonable request.

## Endnotes

^1^
Throughout this manuscript, we use the term EF for brevity. Our discussion is centred specifically on cognitive flexibility, verbal fluency, inhibition, and motor processing speed, aspects of EF with established empirical support for being inefficient or enhanced in AN.
