# Supplementary material for: Identifying Distinct Latent Profiles of Executive Functioning Among Adolescents and Adults With Anorexia Nervosa and Adolescent Healthy Controls
Source: Eur Eat Disord Rev. 2025 Oct 23;34(2):442–54. doi: 10.1002/erv.70043 (PMC12862544; doi:10.1002/erv.70043)
Supplement: Supplementary file 1 — Table S1: Means and Standard Deviations of D‐KEFS Subtests Scaled Scores by Diagnostic Age Group. [file ERV-34-442-s001.docx]

S Table 1. Means and Standard Deviations of D-KEFS Subtests Scaled Scores by Diagnostic Age Group

|  | **Adolescents with AN** | | **Adults with AN** | | **Adolescent HC** | |
| --- | --- | --- | --- | --- | --- | --- |
|  | *M* | *SD* | *M* | *SD* | *M* | *SD* |
| **Trail Making Test Number Letter Switching** | 9.61 | 2.95 | 8.76 | 3.42 | 10.32 | 2.61 |
| **Trail Making Test Motor Speed** | 9.78 | 3.41 | 8.28 | 3.36 | 11.09 | 2.13 |
| **Verbal Fluency Category Switching Total Correct** | 12.38 | 3.70 | 12.79 | 3.87 | 12.47 | 3.09 |
| **Verbal Fluency Category Switching Total Switching Accuracy** | 12.13 | 3.46 | 12.28 | 4.03 | 12.58 | 2.95 |
| **Color Word Interference Inhibition** | 10.93 | 2.89 | 9.17 | 3.93 | 11.32 | 2.26 |
| **Color Word Interference Inhibition/Switching** | 10.67 | 3.06 | 8.59 | 3.85 | 11.19 | 1.84 |
